# Binding of Vialinin A and *p*-Terphenyl Derivatives to Ubiquitin-Specific Protease 4 (USP4): A Molecular Docking Study

**DOI:** 10.3390/molecules27185909

**Published:** 2022-09-11

**Authors:** Christian Bailly, Gérard Vergoten

**Affiliations:** 1 OncoWitan, Consulting Scientific Office, 59290 Lille (Wasquehal), France; 2 Institut de Chimie Pharmaceutique Albert Lespagnol, Faculté de Pharmacie, University of Lille, Inserm, INFINITE-U1286, 3 rue du Professeur Laguesse, BP-83, 59006 Lille, France

**Keywords:** USP4, fungus, molecular modeling, cancer, natural products, anti-inflammatory, molecular docking

## Abstract

The *para*-terphenyl derivative vialinin A (Vi-A), isolated from *Thelephora* fungi, has been characterized as a potent inhibitor of the ubiquitin-specific protease 4 (USP4). Blockade of USP4 contributes to the anti-inflammatory and anticancer properties of the natural product. We have investigated the interaction of Vi-A with USP4 by molecular modeling, to locate the binding site (around residue V98 within the domain in USP segment) and to identify the binding process and interaction contacts. From this model, a series of 32 *p*-terphenyl compounds were tested as potential USP4 binders, mainly in the vialinin, terrestrin and telephantin series. We identified 11 compounds presenting a satisfactory USP4 binding capacity, including two fungal products, vialinin B and aurantiotinin A, with a more favorable empirical energy of USP4 interaction (ΔE) than the reference product Vi-A. The rare *p*-terphenyl aurantiotinin A, isolated from the basidiomycete *T.* *aurantiotincta*, emerged as a remarkable USP4 binder. Structure-binding relationships have been identified and discussed, to guide the future design of USP4 inhibitors based on the *p*-terphenyl skeleton. The docking study should help the identification of other protease inhibitors from fungus.

## 1. Introduction

Ubiquitin-specific protease 4 (USP4) belongs to a family of cysteine proteases responsible for the cleavage of ubiquitin residues on proteins. As a deubiquitinating enzyme (DUB), USP4 is critically implicated in various cellular and biological processes, such as TGF-β response, NFκB signaling and splicing. Its expression and function are tightly regulated under normal physiological conditions. But in different pathological situations, such as inflammatory diseases and cancer, the expression of the enzyme is deregulated, frequently enhanced and uncontrolled. USP4 displays pathological roles in different types of cancers, mainly solid tumors, and several inflammatory diseases, such as asthma [1,2]. This deubiquitinase is considered a target of prime interest to combat growth and dissemination of various tumors, owing to its primary influence on cell proliferation, migration and invasion, and apoptotic cell death [2,3].

**Figure 1:**
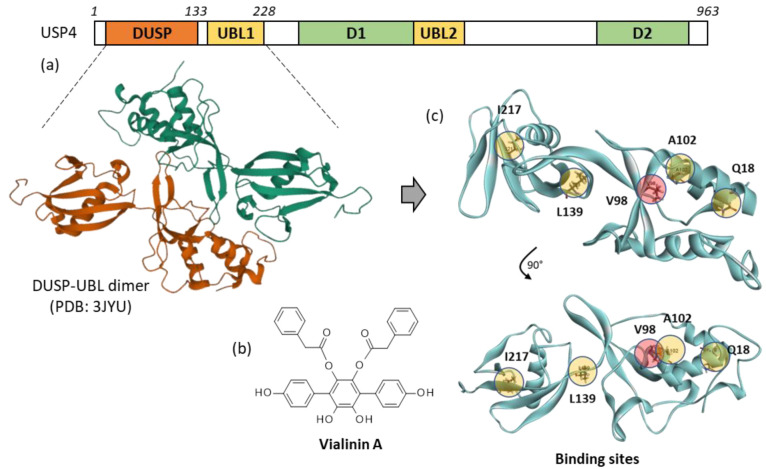
USP4 protein and vialinin binding sites. (**a**) Schematic illustration of the domain organization of USP4, with the domain in USP segment (DUSP), the ubiquitin-like domains (UBL 1 and UBL2) and the domain 1 (D1) and domain 2 (D2) separated by a UBL-containing insert (adapted from Hu et al., 2021). A molecular model of the DUSP-UBL dimer structure of USP4 is shown (from PDB: 3JYU). (**b**) Chemical structure of vialinin A. (**c**) A molecular model of monomeric USP4 with the potential binding sites for vialinin A. Five potential binding sites were identified using the web server CASTp 3.0. The energies of interaction (ΔE) calculated at each site ranked in the order V98 > L139 > Q18 > I217, I102. Site V98 (pointed out in red) was selected for subsequent analyses with Vi-A and analogues.

From a biochemical view, USP4 comprises several adjacent domains, including a domain in USP segment (DUSP) and a ubiquitin-like domain (UBL) (Figure 1a). The protein interacts with many partners, such as the interferon regulatory factor 8 (IRF8), thus stabilizing IRF8 and promoting the suppressive function of regulatory T cells [4,5]. USP4 binds to a variety of effectors, including RNA-binding protein with serine-rich domain 1 (RNPS1) [6], the A2a adenosine receptor [7] and diverse components of the spliceosome assembly [8,9]. The enzyme regulates ubiquitination of many proteins implicated in cancer cells proliferation, such as platelet-derived growth factor β (PDGFβ) [10], and proteins involved in cancer cell stemness, such as Twist 1 [11], to cite only a few examples. USP4 removes conjugated ubiquitin from proteins PDPK1, TRIM21, HAS2, AQP2, TGF-ßR1, and others [12,13]. The deubiquitinase is largely involved in regulation of cancer growth and metastasis, notably in breast cancer [14,15,16], lung cancer [11,17,18], and glioblastoma [19,20]. For these reasons, selective inhibitors of USP4 have been actively searched, to provide research tools and drug candidates potentially useful to combat cancers and inflammatory diseases.

Several inhibitors of USP4, more or less potent and selective, have been discovered in recent years. For example, the dye neutral red has been identified as a non-competitive inhibitor of USP4, capable of reducing the expression and stability of β-catenin, and triggering an anticancer activity in a murine model of colon cancer. This compound is not highly potent toward USP4 (mostly active in the 50–100 μM range) but it presents a selective action, being ineffective toward the related deubiquitinases USP5 and USP14 [17]. More potent USP4 inhibitors have been found, such as chloroacetylpyrrole derivatives with submicromolar activities against USP4 and a good level of USP-selectivity [21]. But the best known, prototypical inhibitor of USP4 is the natural product vialinin A, initially isolated from fruiting bodies of the edible fungus *Thelephora vialis* [22] (Figure 1b). Vialinin A (hereafter designated Vi-A) is a *para*-terphenyl derivative which functions as a powerful inhibitor of TNFα production in rat basophilic leukemia 2H3 (RBL-2H3) cells (IC_50_ = 90 pM) [23]. This fungal product was found to act as an inhibitor of USP enzymes, active against USP4 and USP5 essentially (IC_50_ = 1.5 and 5.9 μM, respectively) and inactive toward USP2 and USP8 [24,25]. Vi-A can be extracted from *T. vialis*, together with other related terphenyl derivatives such as the analogues vialinins B and C [26]. It can be isolated also from the inedible fungus *T. terrestris*, which is at the origin of a series of *p*-terphenyls designated terrestrins. Terrestrin A is a synonym for vialinin A [27]. Chemical procedures have been developed to synthesize vialinins, terrestrins and related *p*-terphenyl compounds [28,29].

Vi-A is a potent inhibitor of the production and release of TNFα in cells, with an efficacy superior to that of the reference immunosuppressor tacrolimus used in organ transplantation. The compound inhibits TNFα translation (not transcription) and modulates proteolysis of pro-inflammatory cytokines [30]. Inhibition of USP4 is considered a key element for the anti-inflammatory action of Vi-A. In a model of autoimmune hepatitis in mice, the expression of USP4 was found to be reduced upon treatment with Vi-A, thereby reducing inflammation and fibrosis in the liver [31]. Importantly, in an animal model of pathological scarring, the treatment with Vi-A produced similar effects to those obtained with a short hairpin RNA for USP4 (shUSP4). USP4 silencing decreased markedly TGFβ-induced cellular proliferation and expression of the TGF-β receptor I, as observed upon treatment with Vi-A. The drug efficiently reduced the formation of pathological scars in the animals [32]. USP4 is a major target for Vi-A.

The unique capacity of Vi-A to bind and inhibit USP4 prompted us to search for other *p*-terphenyl compounds susceptible to interacting with this enzyme. Over the past 15 years, many *p*-terphenyls have been identified and isolated from fungi, principally from the genus *Thelephora*. Various series of *p*-terphenyl derivatives have been characterized, including interesting bioactive compounds with marked anticancer and anti-inflammatory properties, as reviewed recently [33,34]. These considerations prompted us to investigate the capacity of different naturally-occurring *p*-terphenyls to bind to USP4 by means of molecular modeling. We took advantage of the crystal structure of USP4 to establish a model of the USP4/Vi-A complex, with the binding site identified. Then, we compared the binding capacity of 32 *p*-terphenyl derivatives with the objective to identify other potential USP4 binders. A few compounds with an USP4 binding capacity comparable to Vi-A were discovered. Structure-binding relationships are discussed. This computational study will help the characterization and design of novel USP4 inhibitors.

## 2. Results

The tridimensional structure of the N-terminal domains of USP4 has been solved by X-ray crystallography (Protein Data Bank (PDB) access code: 3JYU). We used this structure to perform a docking study to locate the potential binding site for Vi-A. An in silico model of the Vi-A-USP4 complex was elaborated and five potential binding sites were identified using the CASTp 3.0 software. These sites were located around amino acid residues Gln18, Val98, Ala102, Leu139 and Ile217, as shown in Figure 1c (Q18, V98, A102, L139, I217). At each potential site, the empirical energy of interaction (ΔE) was calculated. The best site was found at V98 (ΔE = −113.15 kcal/mol) in a relatively central position of the protein, surrounded by an α-helix and a β-sheet (Figure 1c). The ΔE values calculated for the other sites were significantly less favorable (ΔE = −78.10 kcal/mol at L139, −77.95 kcal/mol at Q18; −72.70 kcal/mol at I217; and −103.10 kcal/mol at A102). Therefore, we retained site V98 for the subsequent analysis with Vi-A and analogues. This site overlaps with A102.

**Figure 2:**
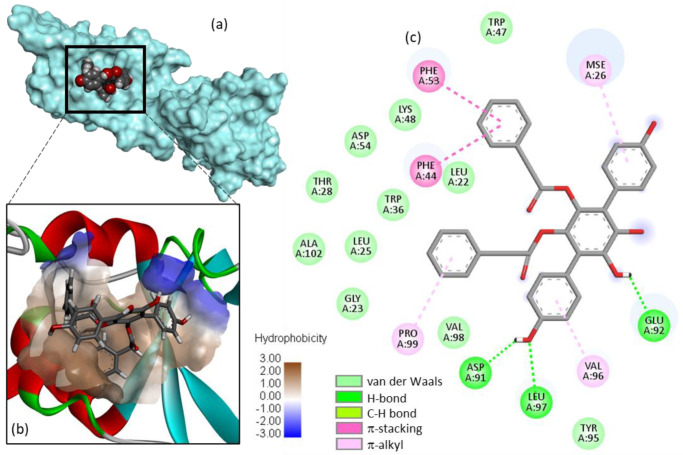
Molecular model of vialinin A (Vi-A) bound to USP4. (**a**) Surface model of Vi-A bound to the V98 site. (**b**) A close-up view of the compound in the binding site, with the hydrophobic/hydrophilic zones colored in brown and blue, respectively. (**c**) Binding map contact, with the indicated color code.

A detailed model of Vi-A bound to USP4 is presented in Figure 2. The compound inserts into a hydrophobic cavity of the protein, with its *p*-terphenyl unit located deep into a small protein groove. A series of molecular contacts maintains the compound into the pocket, stabilized via an array of H-bonds, van der Waals contacts and π-alkyl/π-stacking interactions. Importantly, two phenolic OH of Vi-A are implicated in H-bonds with the protein, via residues Asp-91, Glu-92, and Leu-97. On the other side of the extended molecule, π-stacking interactions with residues Phe-44 and Phe-53 contribute to the stability of the ligand-protein complex. Altogether, about 20 contacts points between Vi-A and USP4 stabilize the complex, providing thus a solid anchorage of the natural product onto the protein surface. The proposed model accounts well for the capacity of Vi-A to inhibit USP4 activity.

**Figure 3:**
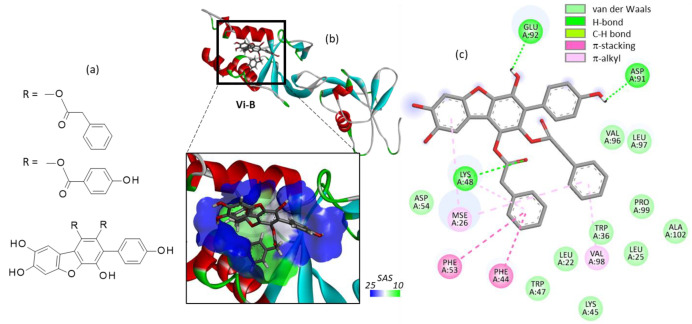
Structures of vialinins B and C. Molecular model of vialinin B (Vi-B) bound to USP4. (**a**) Ribbon model of Vi-B bound to the V98 site. (**b**) A close-up view of the compound in the binding site, with the solvent accessible surface (SAS) colored (color code indicated). (**c**) Binding map contact, with the indicated color code.

Three vialinin derivatives, designated Vi-A, B and C, have been isolated from the edible fungus *Thelephora vialis*. Vi-A bears a *p*-terphenyl unit whereas Vi-B and Vi-C possess a dibenzofuran core [22,26,35]. The three compounds were docked onto the USP4 protein. Interestingly, we found that Vi-B could form more stable complexes with the protein than Vi-A and Vi-C (Table 1). The latter compound bears two *p*-hydroxybenzoyloxy groups on the dibenzofuran core in place of the phenylacetoxy groups present in Vi-A and Vi-B, but the additional phenolic OH of Vi-C apparently does not reinforce the protein binding process. The conformation of Vi-B seems to be better adapted for USP4 binding than Vi-A, but the molecular contacts implicated in the interaction are very similar (Figure 3). Our modeling analysis suggests that Vi-B could be an inhibitor of USP4 as efficient as Vi-A, if not superior. This observation prompted us to investigate other *p*-terphenyl natural products from a fungal origin.

**Figure 4:**
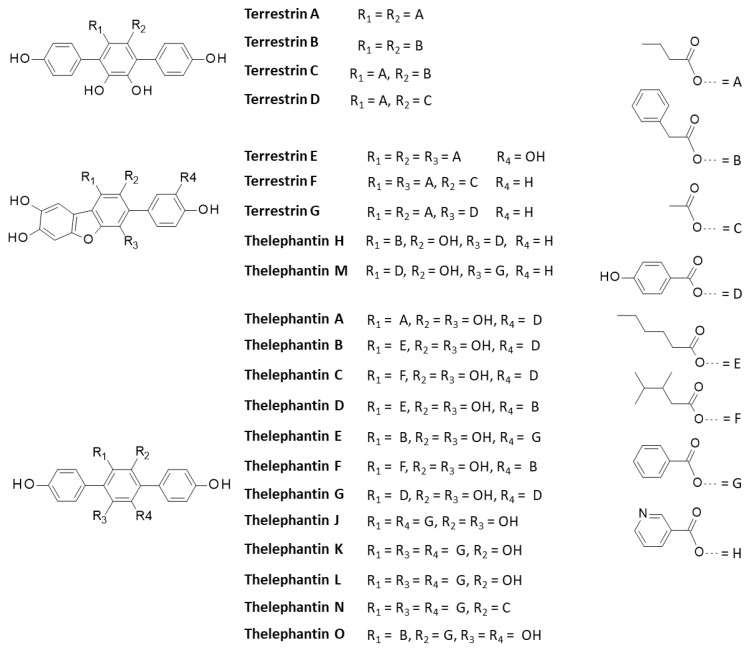
Structures of terrestrins A-G and telephantins A-O. For clarity, the quinone derivative telephantin I is not presented here (it can be found in [33]).

More than 200 *p*-terphenyl compounds have been isolated from fungus [33,36]. We selected 28 additional compounds mainly derived from the parent product terphenyllin and belonging to the thelephantin and terrestrin series (Table 1 and Figure 4). Several of these compounds present interesting anti-inflammatory and/or anticancer activities, as discussed recently [34]. They were all docked onto the V98 site of USP4 to compare with the binding capacity of the vialinins. In each case, the energy of interaction (ΔE) was calculated (Table 1). In the terrestrin series, none of the compounds provided protein complexes more stable than that observed with Vi-A (synonym for terrestrin A). The replacement of one or both of the phenylacetoxy groups of Vi-A with butoyloxy groups (as in terrestrin B) does not improve protein binding. However, it is interesting to note that compounds like terrestrins F and G bearing a dibenzofuran core can form USP4 complexes almost as stable as those observed with Vi-A, but not the tetra-phenolic derivative terrestrin E.

**Figure 5:**
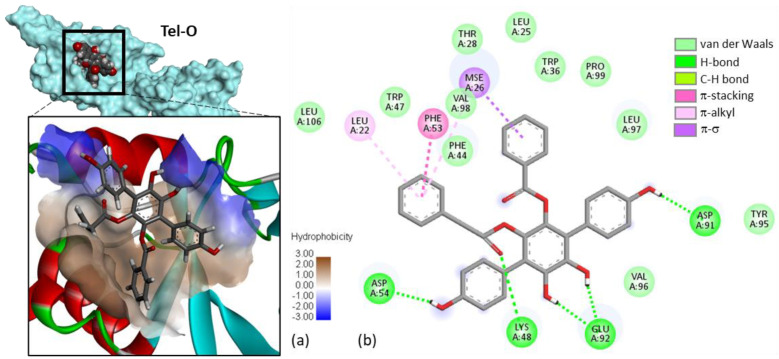
Docking model of telephantin O (Tel-O) bound to USP4. (**a**) Surface model of Tel-O bound to the V98 site, with a close-up view of the compound in the binding site, and the hydrophobic/hydrophilic zones colored in brown and blue, respectively. (**b**) Binding map contact, with the indicated color code.

In the telephantin series, none of the compounds provided more stable complexes with USP4 than Vi-A, but important variations were observed between the various natural products. The case of telephantin O can be underlined because this compound, structurally close to Vi-A, appeared to form complexes with USP4 almost equally stable compared to the parent product (Figure 5). The two phenolic OH groups on the central ring of the *p*-terphenyl unit were found to be engaged in H-bonds with residue Glu-92. The same H-bonding system was observed with Vi-A and Vi-B. The two phenolic OH on the central phenyl ring of telephantin O are apparently key elements for protein binding. The blockade of this phenolic/catechol system, as in telephantin L, markedly reduced the extent of binding. Three other derivatives provided also relatively good binding: telephantins F, H and M with ΔE < −100 kcal/mol (Table 1). The case of telephantin O is interesting because it is the only compound for which we observed a π-σ interaction between the drug and the selenomethionine (SeMet) residue at position 26 of USP4, in the DUSP segment. Similar binding energies (ΔE) were calculated for telephantins O and M, despite their different molecular structure. Telephantin O is a *p*-terphenyl comparable to the vialinin series, whereas telephantin M bears a dibenzofuran tricycle. The binding site can accommodate these two different ligands. In fact, we noted that in the binding cavity, the *p*-terphenyl unit is relatively planar, adopting an extended conformation comparable to the planar dibenzofuran core unit. Moreover, the two compounds present the same phenol group on one side of the molecule and in both cases, this phenol establishes a key H-bond interaction with a aspartic acid residue. In other words, their chemical structure is distinct but their binding configuration is relatively similar. The similarity is interesting and can be exploited to delineate a pharmacophore model composed of a semi-planar biphenyl linked to a third coaxial phenyl unit. In addition, the *p*-terphenyl must be surmounted by a benzyl or similar group.

**Figure 6:**
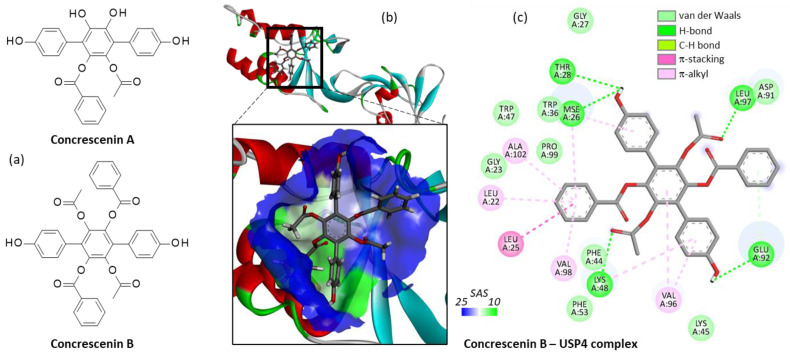
(**a**) Structure of concrescenins A and B. (**b**) Ribbon model of concrescenin B bound to the V98 site of USP4, with a close-up view of the compound in the binding site, and the solvent accessible surface (SAS) colored (color code indicated). (**c**) Binding map contact, with the indicated color code.

Next, we tested two other *p*-terphenyl compounds: concrescenins A and B, both isolated from the mushroom *Hydnellum concrescens* [37]. Surprisingly, we found that the less substituted compound concrescenin A did not bind well to USP4 whereas the bulkier analogue concrescenin B could form stable complexes with the protein, as shown in Figure 6. In this case, the *p*-terphenyl unit was oriented vertically within the binding site, in a fashion significantly distinct from that obtained with Vi-A. It would be interesting to compare the USP4 inhibitory capacity of these products which have been presented as α-glucosidase inhibitors [37], but never described as potential modulators of USP enzymes.

**Figure 7:**
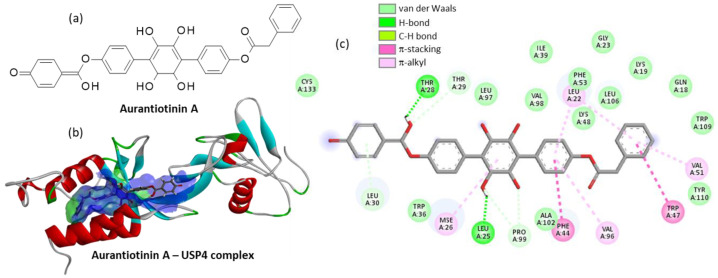
(**a**) Structure of aurantiotinin A. (**b**) Ribbon model of aurantiotinin A bound to the V98 site of USP4, with the solvent accessible surface (SAS) colored. (**c**) Binding map contact, with the indicated color code.

Finally, we tested a few other *p*-terphenyl molecules from a natural origin, such as terphenyllin, terferol, thelephorin A, atromentin, and also the synthetic molecule designated DMT (5′,6′-dimethyl-1,1′:4′,1″-terphenyl-2′,3′,4,4″-tetraol). This latter compound lacking the two phenylacetoxy groups of Vi-A revealed a very weak USP4 binding capability (Table 1). The other compounds were not very good binders with the noticeable exception of the little-known compound called aurantiotinin A. This natural product was isolated twenty years ago from the edible basidiomycete *Thelephora aurantiotincta* Corner [38] but its bioactivity remains totally unknown. Our modeling analysis suggests that the molecule could bind very well to USP4, and thus it could behave as a potent inhibitor, similar to Vi-A. Apparently, aurantiotinin A can form stable complexes with USP4 (ΔE = −118.40 kcal/mol), covering a large surface in the V98 site, due to the extended conformation of the linear molecule (Figure 7). The drug-protein complex is stabilized by a large diversity of molecular contacts, with no less than 25 contacts identified, including H-bonds, van der Waals contacts and π-alkyl/π-stacking interactions (Figure 7c). This is the best compound identified in our series of 32 *p*-terphenyl molecules tested as USP4 binder. Altogether, we found 11 compounds with a noticeable USP4 binding capacity (ΔE = <−100 kcal/mol), including two compounds behaving as better USP4 binders than the reference product Vi-A: Vi-B and aurantiotinin A. This latter natural product deserves a priority investigation as an inhibitor of ubiquitin proteases.

## 3. Discussion and Perspectives

For the first time, a molecular modeling analysis has been performed to locate the interaction site for the *p*-terphenyl fungal product vialinin A bound to the ubiquitin-specific protease USP4. The compound fits into a small groove centered around residue V98 in the DUSP domain of the protein. This domain is essential to the catalytic activity of the protease. It functions as an enhancer of ubiquitin dissociation, and a major allosteric regulator of the active site [39]. Our in silico analysis suggests that the potent USP4 inhibitor Vi-A acts via binding to this N-terminal domain. Based on this model, a few other potential terphenyl-based inhibitors of USP4 have been identified, notably the related natural fungal products vialinin B, telephantins O and M, concrescenin B and the rare compound aurantiotinin A. The latter product has been isolated from the basidiomycete *Thelephora aurantiotincta* which contains numerous *p*-terphenyl products [38]. In fact, many of the vialinin and telephantin derivatives, including the USP4 binders Vi-A, telephantins F, H, O and M have been isolated from this edible fungus [40,41,42,43]. It represents a useful source of bioactive terphenyls.

In recent years, USP4 has gained interest as an anticancer target, contributing to cancer cell proliferation and invasion [9]. In patients with lung adenocarcinoma, an elevated level of USP4 transcripts has been clearly associated with a reduced survival [44]. The protease has been shown to promote the invasive properties of lung tumor cells and stem cells [11,45]. It is therefore interesting to develop USP4 inhibitors as anticancer agents and for this purpose, the *p*-terphenyl skeleton is attractive as a drug design scaffold. These *p*-terphenyls can be obtained by total synthesis [29,46] or extracted from various fungi. Our study provides the necessary basis for further examination of the capacity of *p*-terphenyl to function as USP4 inhibitors. It will be important to investigate further the potency and selectivity of the compounds identified here. Clearly, the *p*-terphenyl moiety should be further exploited in drug design, notably via processes and products inspired by plants and fungus.

We are aware of the limitations posed by in silico studies. The work identifies a few terphenyl derivatives as potential USP4 binders. The drug-USP4 model has been built on the basis of the known interaction of vialinin A with USP4. It is reasonable to consider that the other *p*-terphenyl compounds can bind to the same site but an experimental validation will be necessary. Nevertheless, the work is extremely useful to select the best candidates and to prioritize the syntheses or the isolation of these rare products from fungi. The models can help to design new molecules as well. Experimental binding studies are now required, together with in vivo studies to determine the activity/toxicity ratio for the selected molecules. Vialinin A displays marked antioxidant and antiangiogenic properties in vivo [47] and other *p*-terphenyl compounds have revealed interesting properties in vivo [48,49,50]. It will be useful to investigate further the properties of vialinin B, telephantins O and M, and concrescenin B in animal models.

## 4. Materials and Methods

### 4.1. Molecular Structures and Software

The three-dimensional structure of the USP4 DUSP-UBL dimer (The N-terminal domain of USP4, as represented in Figure 1a) was retrieved from the Protein Data Bank (www.rcsb.org, accessed on 1 September 2022) under the PDB code 3JYU. It is a convenient, well-resolved (2.37 Å) crystallized protein structure to identify USP-binding ligands [51]. Docking experiments were performed using the GOLD software (GOLD 5.3 release, Cambridge Crystallographic Data Centre, Cambridge, UK). Molecular graphics and analyses were performed using Discovery Studio Visualizer, Biovia 2020 (Dassault Systèmes BIOVIA Discovery Studio Visualizer 2020; San Diego, Dassault Systèmes, 2020).

### 4.2. In Silico Molecular Docking Procedure

The process used includes the following five steps.

(1) Monte Carlo (MC) conformational search of the ligand using the BOSS (Biochemical and Organic Simulation System) software, freely available to academic users. The structure of each ligand was optimized using a classical MC conformational search procedure, as described in BOSS [52]. A conformational analysis has been performed to define the best starting geometries for each compound. An energy minimization was carried out to identify all minimum-energy conformers, leading to the identification of a unique conformer for the free ligand. Within BOSS, MC simulations were performed in the constant-temperature and constant-pressure ensemble (NPT).
(2) Evaluation of the free energy of hydration for the chosen structure of the ligand. The molecular mechanics/generalized Born surface area (MM/GBSA) procedure was used to evaluate the free energies of hydration (ΔG) [53]. ΔG is directly related to solubility of the compound [54]. MC search and computation of ΔG were performed within BOSS using the xMCGB script according to procedures given in references [53,55]. The best ligand structure is then used in the docking procedure.
(3) Determination of the protein-ligand site of interaction. Potential M^pro^-binding sites for the different products were searched using the web server CASTp 3.0 (Computed Atlas of Surface Topography of proteins) and visualized with the molecular modeling system Chimera 1.15 [56,57]. Shape complementarity and geometry considerations favor a docking grid centered in the volume defined by the central amino acid. Within the binding site, the side chains of the specific amino acids were considered fully flexible during docking. With the 3JYU structure, based on shape complementarity criteria, the best USP4 binding site for the ligands has been defined around amino acid residue Val98 (see Results below) and the flexible amino acids are Trp43, Phe44, Trp47, Lys48, Phe53, Glu92, Val96, Val98, Trp103, and Trp109.
(4) Docking procedure using GOLD. In our typical docking process, 100 energetically reasonable poses (according to the ChemPLP scoring function) are retained while searching for the correct binding mode of the ligand. The decision to maintain a trial pose is based on ranked poses, using the PLP fitness scoring function (which is the default in GOLD version 5.3 used here) [58]. Six poses are kept. The empirical potential energy of the interaction ΔE for the ranked complexes was evaluated using the simple expression ΔE(interaction) = E(complex) − [E(protein) + E(ligand)]. Calculations of the final energy are performed on the basis of the SPASIBA spectroscopic force field. The corresponding parameters are derived from vibrational wavenumbers obtained in the infrared and Raman spectra of a large series of compounds including organic molecules, amino-acids, saccharides, nucleic acids and lipids.
(5) Validation using the SPASIBA force field. This last step is considered essential to define the best protein-ligand structure. The spectroscopic SPASIBA (Spectroscopic Potential Algorithm for Simulating Biomolecular conformational Adaptability) force field has been specifically developed to provide refined empirical molecular mechanics force field parameters [59]. It is a spectroscopic molecular-mechanics-derived empirical potential function first devoted to proteins and extended to a large variety of chemical groups (including amino acids, saccharides, and phospholipids). This force field is a very appropriate tool for molecular mechanics which is able to reproduce at the same time structures, energies and vibrational spectra with a much higher precision on the latter than commonly available force fields. SPASIBA introduces the Urey–Bradley F quadratic force constant related to the 1–3 nonbonded distance in a bond angle, solves the redundancy problem among internal coordinates, and adds specific parameters to correctly fit spectra [60,61]. SPASIBA (integrated into CHARMM) [62] has been shown to be excellent for reproducing crystal phase infrared data. The same procedure was used to establish molecular models for the various natural products listed in Table 1, including the reference product Vi-A.

## 5. Conclusions

The binding site for Vi-A bound to the protease USP4 has been located around position V98 in the DUSP domain. The phenolic hydroxyl groups on the *p*-terphenyl skeleton are essential to anchor the compound onto the binding cavity. Molecular modeling predicts that Vi-A but also Vi-B can form stable complexes with USP4. A few other *p*-terphenyl natural products of fungal origin have been identified as potential USP4 binders, notably telephantins O and M, concrescenin B and aurantiotinin A. The study will guide the design of *p*-terphenyl-based USP4 inhibitors of interest as anti-inflammatory and anticancer agents.

## Figures and Tables

**Table 1:** Calculated potential energy of interaction (ΔE, kcal/mol) for the interaction of terphenyl compounds with USP4 (PDB: 3JYU).

Compound	CID#^a^	ΔE
Atromentin	99148	−74.93
Aurantiotinin A	(*)	−118.40
Concrescenin A	139584566	−76.79
Concrescenin B	139587861	−109.22
DMT	(*)	−64.45
Terferol	167947	−79.50
Terphenyllin	100437	−75.24
Terrestrin B	60202116	−87.55
Terrestrin C	101743853	−88.55
Terrestrin D	60201958	−89.62
Terrestrin E	101743854	−62.05
Terrestrin F	101743855	−102.70
Terrestrin G	101743856	−103.65
Thelephantin A	5321927	−79.97
Thelephantin B	5321928	−92.13
Thelephantin C	5321929	−76.47
Thelephantin D	101260622	−96.31
Thelephantin E	139583228	−78.31
Thelephantin F	10126023	−103.24
Thelephantin G	101245355	−87.40
Thelephantin H	101260624	−102.10
Thelephantin I	21577381	−82.80
Thelephantin J	21577382	−72.04
Thelephantin K	21577383	−84.50
Thelephantin L	21577384	−70.92
Thelephantin M	(*)	−104.40
Thelephantin N	21577386	−92.40
Thelephantin O	53375718	−105.70
Thelephorin A	10325564	−98.47
Vialinin A	11563133	−108.06
Vialinin B	16049791	−116.76
Vialinin C	86279343	−106.26

^a^ Compound Identity number, as defined in PubChem (https://pubchem.ncbi.nlm.nih.gov, accessed on 1 September 2022). Refer to [33] for the structures of all compounds. (*) These compounds are not listed in the PubChem databank.

## Data Availability

Not applicable.

## Sample Availability

Not available.
